# Supplementary material for: Mechanistic computational modeling of monospecific and bispecific antibodies targeting interleukin-6/8 receptors
Source: PLoS Comput Biol. 2024 Jun 7;20(6):e1012157. doi: 10.1371/journal.pcbi.1012157 (PMC11189202; doi:10.1371/journal.pcbi.1012157)
Supplement: S1 File — The ordinary differential equations (ODEs) used for modeling tocilizumab, 10H2, and BS1 binding interactions with IL-6R and IL-8R. (PDF) [file pcbi.1012157.s001.pdf]

Binding Model Equations

α is a unit conversion parameter, used in Equations S2, S5, S8 to convert the rate terms from (# receptors per cell)$^{-1}$ s^{-1} to nM^{-1} s^{-1}. It is defined in Equation S1 where n_{cell} is the number of cells in the experiment or simulation, V is the volume containing the cells and the antibodies, and N_A is Avogadro's number, 6.022 × 10^{23} molecules per mole. For the conditions used in the flow cytometry binding assays and in the model simulations, n_{cell} = 10^5 cells and V = 200 μL, making α = 8.3 × 10^{-7} nM/(# / cell).

$$\alpha = \frac{n_{cell}}{V * N_A} \qquad (S1)$$

$$\frac{d[Toci]}{dt} = \alpha * k_{off,Toci-6R}[Toci \cdot 6R] - 2 * \alpha * k_{on,Toci-6R}[Toci][6R] \qquad (S2)$$

$$\frac{d[Toci \cdot 6R]}{dt} = 2 * k_{on,Toci-6R}[Toci][6R] + 2 * k_{off,Toci-6R^*}[6R \cdot Toci \cdot 6R] - k_{on,Toci-6R^*}[6R][Toci \cdot 6R] - k_{off,Toci-6R}[Toci \cdot 6R] \qquad (S3)$$

$$\frac{d[6R \cdot Toci \cdot 6R]}{dt} = k_{on,Toci-6R^*}[6R][Toci \cdot 6R] - 2 * k_{off,Toci-6R^*}[6R \cdot Toci \cdot 6R] \qquad (S4)$$

$$\frac{d[H2]}{dt} = \alpha * k_{off,H2-8R}[H2 \cdot 8R] - 2 * \alpha * k_{on,H2-8R}[H2][8R] \qquad (S5)$$

$$\frac{d[H2 \cdot 8R]}{dt} = 2 * k_{on,H2-8R}[H2][8R] + 2 * k_{off,H2-8R^*}[8R \cdot H2 \cdot 8R] - k_{on,H2-8R^*}[8R][H2 \cdot 8R] - k_{off,H2-8R}[H2 \cdot 8R] \qquad (S6)$$

$$\frac{d[8R \cdot H2 \cdot 8R]}{dt} = k_{on,H2-8R^*}[8R][H2 \cdot 8R] - 2 * k_{off,H2-8R^*}[8R \cdot H2 \cdot 8R] \qquad (S7)$$

$$\frac{d[BS1]}{dt} = \alpha * k_{off,BS1-6R}[BS1 \cdot 6R] + \alpha * k_{off,BS1-8R}[BS1 \cdot 8R] - \alpha * k_{on,BS1-6R}[BS1][6R] - \alpha * k_{on,BS1-8R}[BS1][8R] \qquad (S8)$$

$$\begin{aligned}\frac{d[BS1\cdot 6R]}{dt} &= k_{on,BS1-6R}[BS1][6R] + k_{off,BS1-8R^*}[6R\cdot BS1\cdot 8R] \\ &\quad -k_{on,BS1-8R^*}[8R][BS1\cdot 6R] - k_{off,BS1-6R}[BS1\cdot 6R]\end{aligned} \qquad (S9)$$

$$\begin{aligned}\frac{d[BS1\cdot 8R]}{dt} &= k_{on,BS1-8R}[BS1][8R] + k_{off,BS1-6R^*}[6R\cdot BS1\cdot 8R] \\ &\quad -k_{on,BS1-6R^*}[6R][BS1\cdot 8R] - k_{off,BS1-8R}[BS1\cdot 8R]\end{aligned} \qquad (S10)$$

$$\begin{aligned}\frac{d[6R\cdot BS1\cdot 8R]}{dt} &= k_{on,BS1-6R^*}[6R][BS1\cdot 8R] + k_{on,BS1-8R^*}[8R][BS1\cdot 6R] \\ &\quad -k_{off,BS1-6R^*}[6R\cdot BS1\cdot 8R] - k_{off,BS1-8R^*}[6R\cdot BS1\cdot 8R]\end{aligned} \qquad (S11)$$

$$\begin{aligned}\frac{d[6R]}{dt} &= k_{off,Toci-6R}[Toci\cdot 6R] + 2 * k_{off,Toci-6R^*}[6R\cdot Toci\cdot 6R] \\ &\quad +k_{off,BS1-6R}[BS1\cdot 6R] + k_{off,BS1-6R^*}[6R\cdot BS1\cdot 8R] \\ &\quad -2 * k_{on,Toci-6R}[Toci][6R] - k_{on,Toci-6R^*}[6R][Toci\cdot 6R] \\ &\quad -k_{on,\text{[illegible]}S1-6R}[BS1][6R] - k_{on,BS1-6R^*}[6R][BS1\cdot 8R]\end{aligned} \qquad (S12)$$

$$\begin{aligned}\frac{d[8R]}{dt} &= k_{off,H2-8R}[H2\cdot 8R] + 2 * k_{off,H2-8R^*}[8R\cdot H2\cdot 8R] \\ &\quad +k_{off,BS1-8R}[BS1\cdot 8R] + k_{off,BS1-8R^*}[6R\cdot BS1\cdot 8R] \\ &\quad -2 * k_{on,H2-8R}[H2][8R] - k_{on,H2-8R^*}[8R][H2\cdot 8R] \\ &\quad -k_{on,BS1-8R}[BS1][8R] - k_{on,BS1-8R^*}[8R][BS1\cdot 6R]\end{aligned} \qquad (S13)$$
