# Supplementary material for: Mechanistic computational modeling of monospecific and bispecific antibodies targeting interleukin-6/8 receptors
Source: PLoS Comput Biol. 2024 Jun 7;20(6):e1012157. doi: 10.1371/journal.pcbi.1012157 (PMC11189202; doi:10.1371/journal.pcbi.1012157)
Supplement: S2 File — Description of the different schemes used for normalizing the simulation output for comparison with the in vitro experimental data. (PDF) [file pcbi.1012157.s002.pdf]

Normalization Schemes

Optimization of the binding model rate constants with the different normalization schemes (detailed in **Table 3**) yields similar optimized parameter sets, with slightly different parameter value distributions for each scheme [**S4 Fig**]. For the optimizations normalized to the BS1 data at binding saturation in particular, the optimizations converged to a single parameter set at a very high frequency [**S4A Fig**].

While the optimizations with normalization against the individual antibodies do still contain a most frequent optimal point, they also yield a wider variety of possible parameter values [**S4B Fig**]. Each of the distributions shows two or more commonly reoccurring values, and the lowest cost parameter value is not always the same as the most frequent value. Some of the parameter values are similar to the values from the normalization against BS1, but the $k_{on,6R^*}$ and the $k_{off,8R}$ values differ by almost two orders of magnitude.

Further, optimizations performed with normalization against the BS1 data, both normalized to the bound concentrations at binding saturation and normalized to the maximum bound concentration, demonstrate a greater proportion of low-cost optimal sets than the optimizations normalized to each antibody separately, as illustrated by the cumulative distribution of the cost of each set of optimal values [**S5 Fig**]. The greater variance in optimal values for the normalization to individual antibodies separately is likely due to a reduction in data meaningfulness when comparisons between antibodies are lost. Many of the parameter sets from the different normalization schemes match well to the experimental data, but a few sets show more substantial variation [**S7 Fig**].

In total, these results demonstrate that ideal normalization scheme for the model output is normalization to the amount of bound BS1 at binding saturation (i.e., “BS1, Data”). This scheme showed a strong convergence to a single optimal parameter set, and simulations with this optimal parameter set replicate the experimental data well. For these reasons, this normalization scheme was selected as the primary normalization method for the model analysis as described in the *Results*.
