# Supplementary material for: Mechanistic computational modeling of monospecific and bispecific antibodies targeting interleukin-6/8 receptors
Source: PLoS Comput Biol. 2024 Jun 7;20(6):e1012157. doi: 10.1371/journal.pcbi.1012157 (PMC11189202; doi:10.1371/journal.pcbi.1012157)
Supplement: S1 Table — (PDF) [file pcbi.1012157.s003.pdf]

S1 Table. *Original model parameters and their relationship to the simplified parameters after applying the model assumptions.*

**Original**	**Simplified**	**Best Fit Value**	**Units**
$k_{on,Toci-6R}$	$k_{on,6R}$	5.92×10^{-6}	$nM^{-1}s^{-1}$
$k_{on,Toci-6R^*}$	$k_{on,6R^*}$	8.11×10^{-8}	$\left(\frac{\#}{cell}\right)^{-1} s^{-1}$
$k_{off,Toci-6R}$	$k_{off,6R}$	5.61×10^{-5}	s^{-1}
$k_{off,Toci-6R^*}$	$k_{off,6R}$	5.61×10^{-5}	s^{-1}
$k_{on,H2-8R}$	$k_{on,8R}$	9.03×10^{-6}	$nM^{-1}s^{-1}$
$k_{on,H2-8R^*}$	$k_{on,8R^*}$	1.24×10^{-7}	$\left(\frac{\#}{cell}\right)^{-1} s^{-1}$
$k_{off,H2-8R}$	$k_{off,8R}$	6.38×10^{-5}	s^{-1}
$k_{off,H2-8R^*}$	$k_{off,8R}$	6.38×10^{-5}	s^{-1}
$k_{on,BS1-6R}$	$k_{on,6R}$	5.92×10^{-6}	$nM^{-1}s^{-1}$
$k_{on,BS1-8R}$	$k_{on,8R}$	9.03×10^{-6}	$nM^{-1}s^{-1}$
$k_{on,BS1-6R^*}$	$k_{on,6R^*}$	8.11×10^{-8}	$\left(\frac{\#}{cell}\right)^{-1} s^{-1}$
$k_{on,BS1-8R^*}$	$k_{on,8R^*}$	1.24×10^{-7}	$\left(\frac{\#}{cell}\right)^{-1} s^{-1}$
$k_{off,BS1-6R}$	$k_{off,6R}$	5.61×10^{-5}	s^{-1}
$k_{off,BS1-8R}$	$k_{off,8R}$	6.38×10^{-5}	s^{-1}
$k_{off,BS1-6R^*}$	$k_{off,6R}$	5.61×10^{-5}	s^{-1}
$k_{off,BS1-8R^*}$	$k_{off,8R}$	6.38×10^{-5}	s^{-1}
