# Supplementary material for: Mechanistic computational modeling of monospecific and bispecific antibodies targeting interleukin-6/8 receptors
Source: PLoS Comput Biol. 2024 Jun 7;20(6):e1012157. doi: 10.1371/journal.pcbi.1012157 (PMC11189202; doi:10.1371/journal.pcbi.1012157)
Supplement: S2 Table — Values are given as dissociation constants (KD) in nM and were originally reported by Yang et al [28]. (PDF) [file pcbi.1012157.s004.pdf]

S2 Table. *IL-6R and IL-8R binding affinities for the monospecific and bispecific antibodies calculated directly from in vitro HEK 293T cell surface binding assays. Values are given as dissociation constants (K_D) in nM and were originally reported by Yang et al (1).*

	**IL-6R⁺ / IL-8R⁻**	**IL-6R⁻ / IL-8R⁺**	**IL-6R⁺ / IL-8R⁺**
Tocilizumab	3.1	N/A	3.1
10H2	N/A	3.6	4.1
BS1	24	10	14

Reference

1. Yang H, Karl MN, Wang W, Starich B, Tan H, Kiemen A, et al. Engineered bispecific antibodies targeting the interleukin-6 and -8 receptors potently inhibit cancer cell migration and tumor metastasis. Mol Ther 2022;30(11):3430–3449. doi: 10.1016/j.ymthe.2022.07.008.
