# Supplementary material for: Mechanistic computational modeling of monospecific and bispecific antibodies targeting interleukin-6/8 receptors
Source: PLoS Comput Biol. 2024 Jun 7;20(6):e1012157. doi: 10.1371/journal.pcbi.1012157 (PMC11189202; doi:10.1371/journal.pcbi.1012157)
Supplement: S1 Fig — Schematic of the IL-6Rα/IL-8RB antibody-binding model for the two monoclonal antibodies, tocilizumab (anti-IL-6Rα) (A) and 10H2 (anti-IL-8RB) (B). As in the BS1 binding model (Fig 1B), kon,6R and kon,8R describe the association rates for the formation of binary antibody-receptor complexes, and kon,6R* and kon,8R* describe the association rates for the formation ternary receptor-antibody-receptor complexes. The same koff,6R and koff,8R rate constants are used for the dissociation of both the binary and the ternary complexes. Notably, in this study we simplify the parameter optimization by assuming that the binding rate constants for the bispecific antibody (Fig 1B) are the same as the equivalent reactions for the monospecific antibodies. This figure was created with BioRender.com. (PDF) [file pcbi.1012157.s005.pdf]

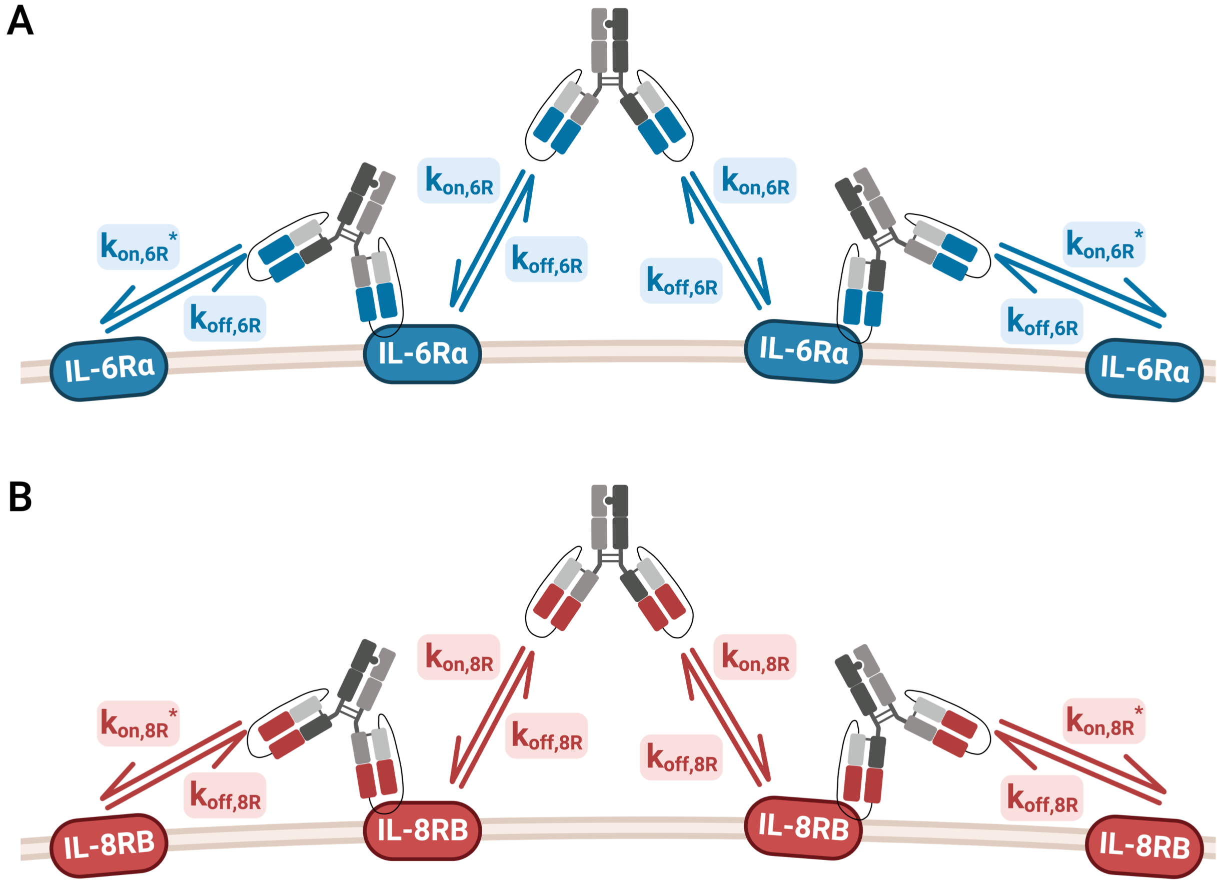

S1 Fig. Monoclonal antibody binding model kinetics. Schematic of the IL-6Rα/IL-8RB antibody-binding model for the two monoclonal antibodies, tocilizumab (anti-IL-6Rα) (**A**) and 10H2 (anti-IL-8RB) (**B**). As in the BS1 binding model [**Fig 1B**], $k_{on,6R}$ and $k_{on,8R}$ describe the association rates for the formation of binary antibody-receptor complexes, and $k_{on,6R^*}$ and $k_{on,8R^*}$ describe the association rates for the formation ternary receptor-antibody-receptor complexes. The same $k_{off,6R}$ and $k_{off,8R}$ rate constants are used for the dissociation of both the binary and the ternary complexes. Notably, in this study we simplify the parameter optimization by assuming that the binding rate constants for the bispecific antibody [**Fig 1B**] are the same as the equivalent reactions for the monospecific antibodies. This figure was created with BioRender.com.
