# Supplementary material for: Mechanistic computational modeling of monospecific and bispecific antibodies targeting interleukin-6/8 receptors
Source: PLoS Comput Biol. 2024 Jun 7;20(6):e1012157. doi: 10.1371/journal.pcbi.1012157 (PMC11189202; doi:10.1371/journal.pcbi.1012157)
Supplement: S2 Fig — BS1 describes simulations that were normalized against the concentration of bound BS1 at the end time point, and Ab indicates simulations that were normalized against the concentration of that specific antibody at the end time point. Data depicts simulations that were normalized using the bound concentrations at the binding saturation, as was done for the experimental data, and Max describes simulations that were normalized using the bound concentration at the maximum initial antibody concentration. (PDF) [file pcbi.1012157.s006.pdf]

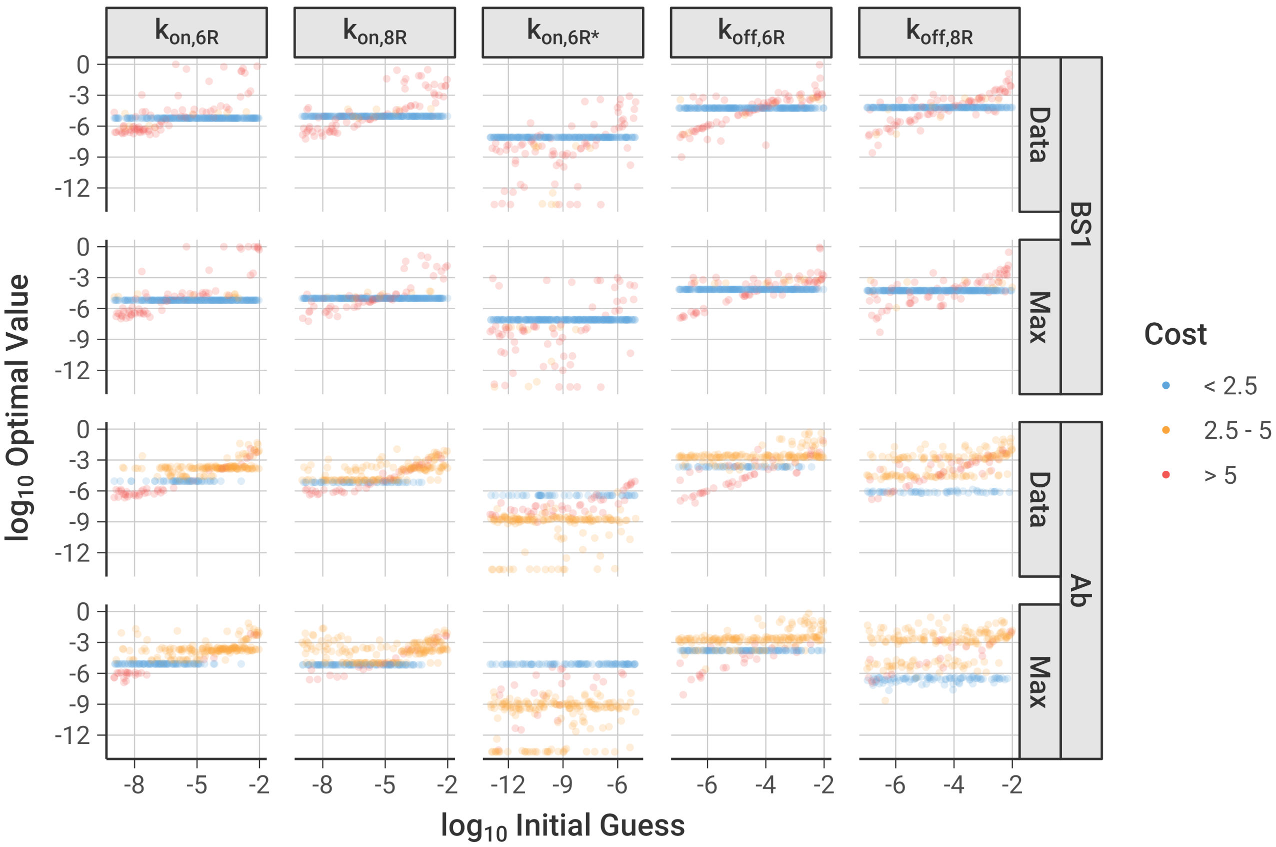

S2 Fig. Relationship between initial guesses and optimized values for each binding reaction rate constant, separated by normalization options used. ***BS1*** describes simulations that were normalized against the concentration of bound BS1 at the end time point, and ***Ab*** indicates simulations that were normalized against the concentration of that specific antibody at the end time point. ***Data*** depicts simulations that were normalized using the bound concentrations at the binding saturation, as was done for the experimental data, and ***Max*** describes simulations that were normalized using the bound concentration at the maximum initial antibody concentration.
