# Supplementary material for: Mechanistic computational modeling of monospecific and bispecific antibodies targeting interleukin-6/8 receptors
Source: PLoS Comput Biol. 2024 Jun 7;20(6):e1012157. doi: 10.1371/journal.pcbi.1012157 (PMC11189202; doi:10.1371/journal.pcbi.1012157)
Supplement: S3 Fig — To better visualize the distribution of the parameter sets, the plotted values are limited to one order of magnitude above and below the values from the lowest cost parameter set (Table 2). A, Distribution of optimized parameter values across all optimizations performed, with marked points indicating the values of the lowest cost parameter set. B, Relationship between optimized parameter values and the cost of the optimized parameter sets compared to experimental data, separated by parameter. Optimized points with the same value are grouped into a single point, with the point size indicating how many optimized parameter values are in the group. (PDF) [file pcbi.1012157.s007.pdf]

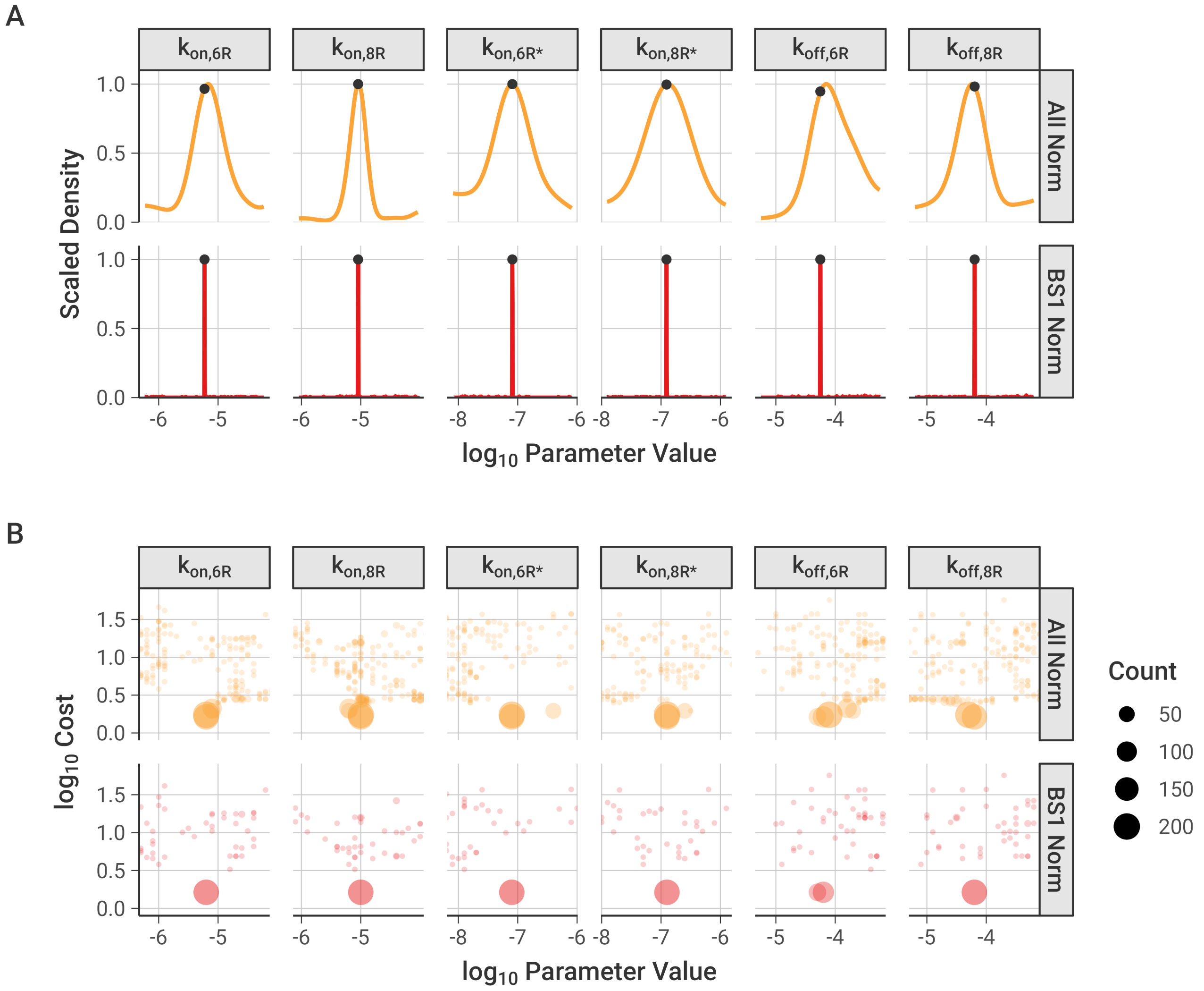

S3 Fig. Frequency and cost of optimized binding model parameter sets, showing a limited range of values around the lowest-cost parameter set. To better visualize the distribution of the parameter sets, the plotted values are limited to one order of magnitude above and below the values from the lowest cost parameter set [**Table 2**]. **A,** Distribution of optimized parameter values across all optimizations performed, with marked points indicating the values of the lowest cost parameter set. **B,** Relationship between optimized parameter values and the cost of the optimized parameter sets compared to experimental data, separated by parameter. Optimized points with the same value are grouped into a single point, with the point size indicating how many optimized parameter values are in the group.
