# Supplementary material for: Mechanistic computational modeling of monospecific and bispecific antibodies targeting interleukin-6/8 receptors
Source: PLoS Comput Biol. 2024 Jun 7;20(6):e1012157. doi: 10.1371/journal.pcbi.1012157 (PMC11189202; doi:10.1371/journal.pcbi.1012157)
Supplement: S4 Fig — Normalization types are separated in the same way as for the initial guesses (S2 Fig). A, Distribution of optimized parameter values across all optimizations performed, separated by normalization options. Marked points and corresponding labels indicate the values of the lowest cost parameter set for those specific normalization options. B, Relationship between optimized parameter values and cost compared to experimental data, separated by parameter. Optimized points with the same value are grouped into a single point, with the point size indicating how many optimized parameters are in the group. (PDF) [file pcbi.1012157.s008.pdf]

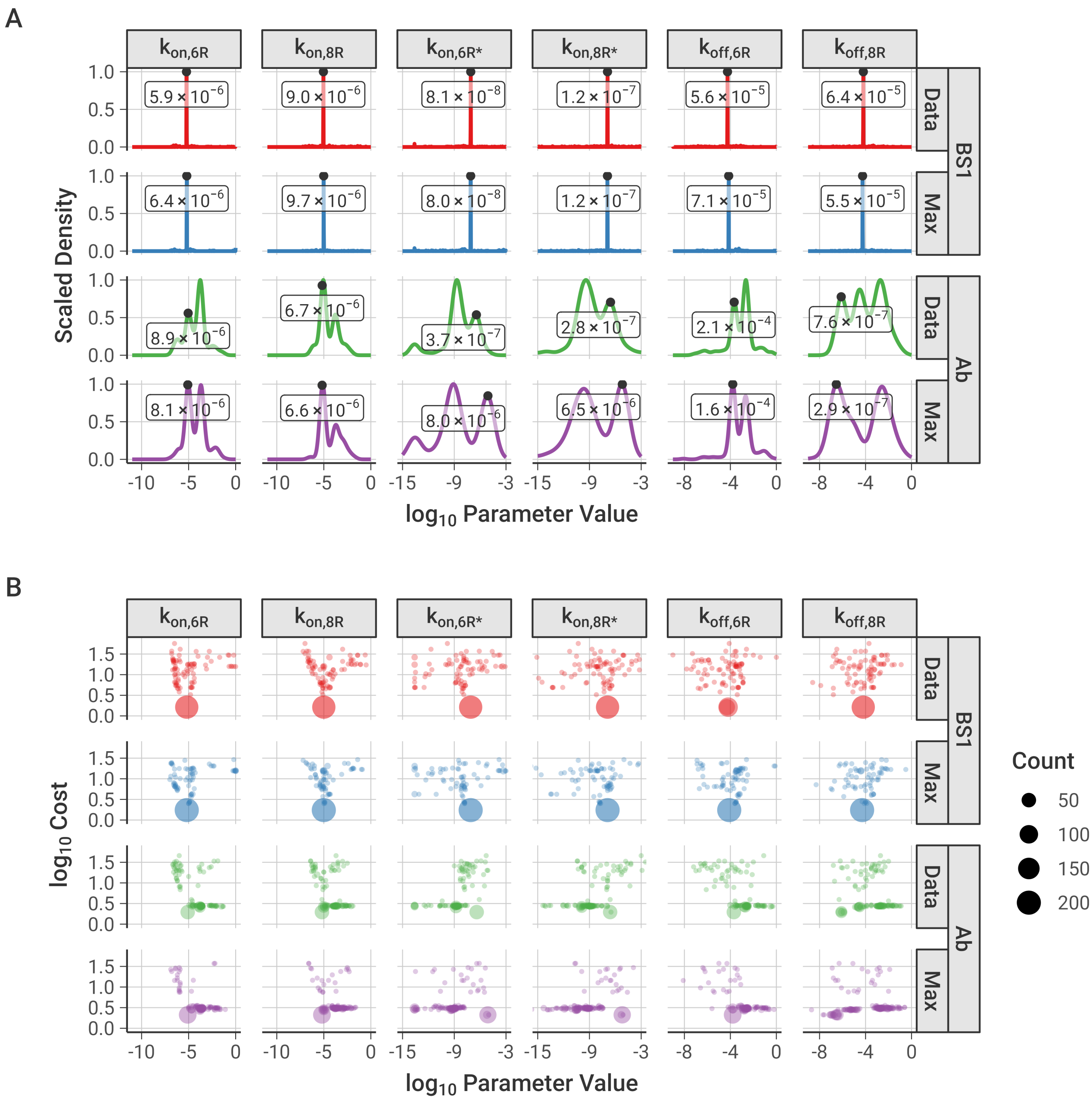

S4 Fig. Distribution of optimized parameter values and cost compared to experimental data, separated by parameter and normalization type. Normalization types are separated in the same way as for the initial guesses [**S2 Fig**]. **A,** Distribution of optimized parameter values across all optimizations performed, separated by normalization options. Marked points and corresponding labels indicate the values of the lowest cost parameter set for those specific normalization options. **B,** Relationship between optimized parameter values and cost compared to experimental data, separated by parameter. Optimized points with the same value are grouped into a single point, with the point size indicating how many optimized parameters are in the group.
