# Supplementary material for: Mechanistic computational modeling of monospecific and bispecific antibodies targeting interleukin-6/8 receptors
Source: PLoS Comput Biol. 2024 Jun 7;20(6):e1012157. doi: 10.1371/journal.pcbi.1012157 (PMC11189202; doi:10.1371/journal.pcbi.1012157)
Supplement: S5 Fig — The curves depict the fraction of optimal parameter sets that were below a given cost value. Parameter sets where the optimization did not converge were omitted. (PDF) [file pcbi.1012157.s009.pdf]

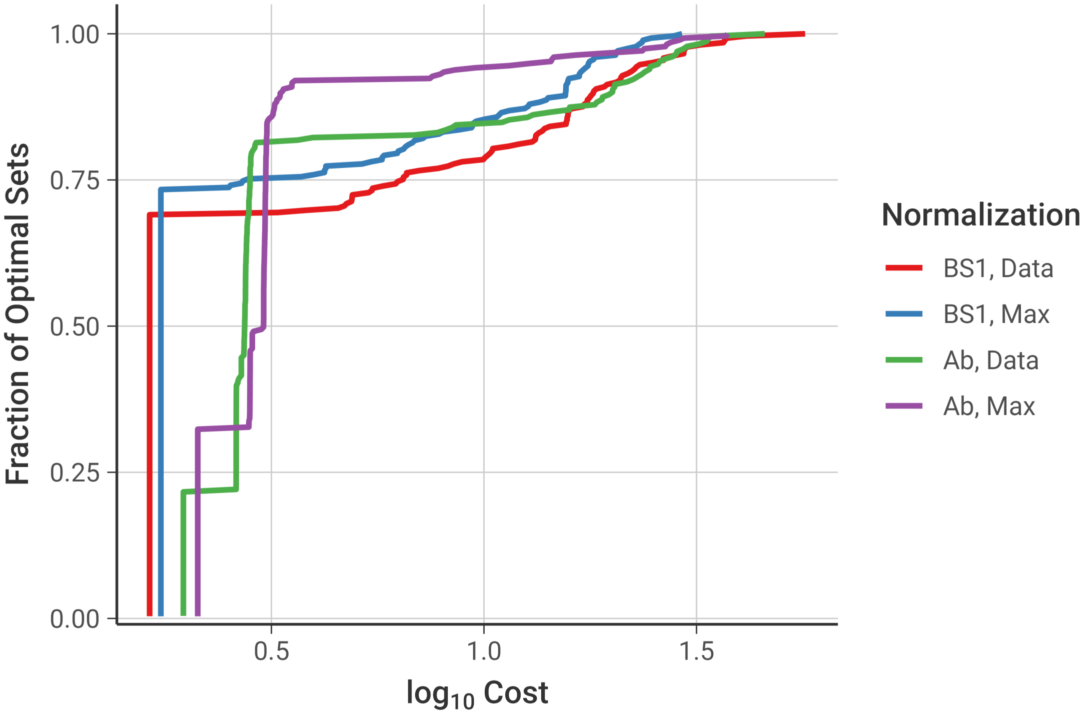

S5 Fig. Cumulative distribution of the cost of the optimized parameter sets, separated by normalization options used. The curves depict the fraction of optimal parameter sets that were below a given cost value. Parameter sets where the optimization did not converge were omitted.
