# Supplementary material for: Mechanistic computational modeling of monospecific and bispecific antibodies targeting interleukin-6/8 receptors
Source: PLoS Comput Biol. 2024 Jun 7;20(6):e1012157. doi: 10.1371/journal.pcbi.1012157 (PMC11189202; doi:10.1371/journal.pcbi.1012157)
Supplement: S6 Fig — Bound concentrations are divided among binary complexes, ternary complexes, and total bound antibody. Simulations were performed under the same conditions as the binding experiments: 105 cells/well, receptor expression levels from the transduced cell lines (Table 1), and with a 2-hour initial association period followed by a 15-minute free antibody washout. Model output is normalized to the bound concentration of BS1 at the same initial antibody concentrations used to normalize the experimental data. (PDF) [file pcbi.1012157.s010.pdf]

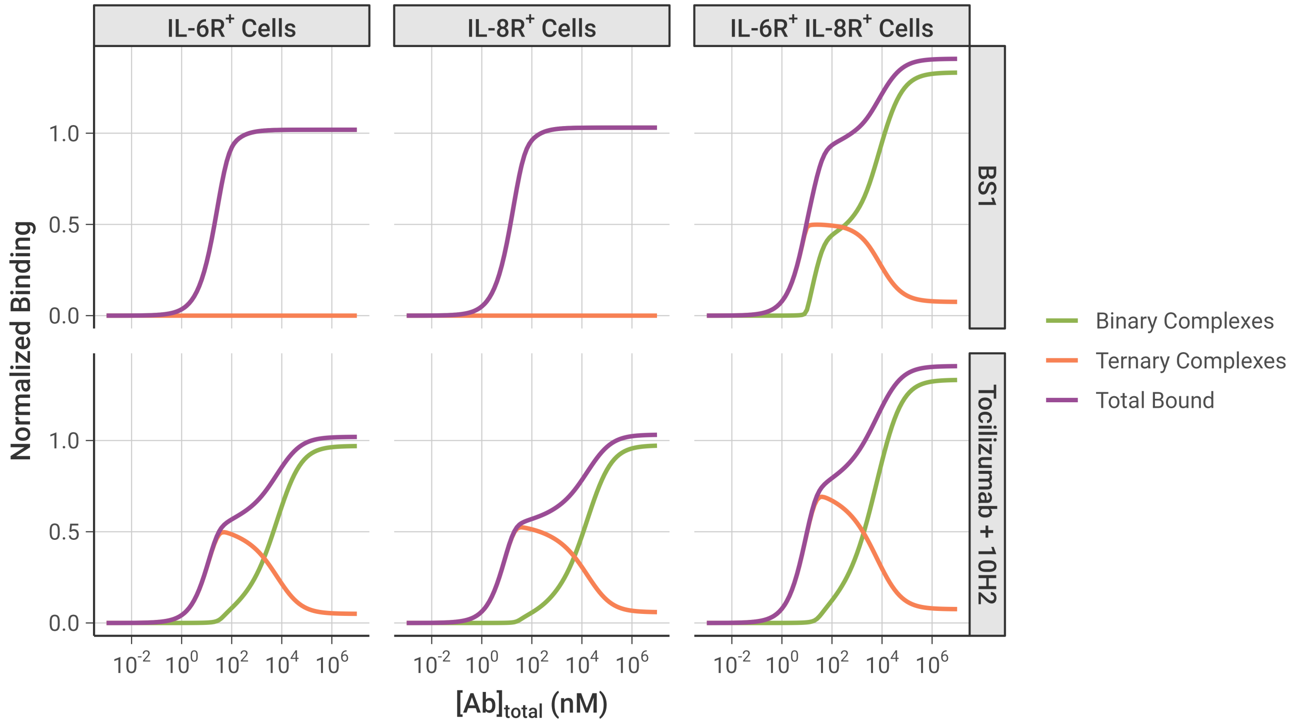

S6 Fig. Comparison of normalized binding curves between the different antibodies. Bound concentrations are divided among binary complexes, ternary complexes, and total bound antibody. Simulations were performed under the same conditions as the binding experiments: 10^5 cells/well, receptor expression levels from the transduced cell lines [**Table 1**], and with a 2-hour initial association period followed by a 15-minute free antibody washout. Model output is normalized to the bound concentration of BS1 at the same initial antibody concentrations used to normalize the experimental data.
