# Supplementary material for: Mechanistic computational modeling of monospecific and bispecific antibodies targeting interleukin-6/8 receptors
Source: PLoS Comput Biol. 2024 Jun 7;20(6):e1012157. doi: 10.1371/journal.pcbi.1012157 (PMC11189202; doi:10.1371/journal.pcbi.1012157)
Supplement: S7 Fig — Simulations were performed under the same conditions as the experiment, with a 2-hour initial binding period followed by a 15-minute antibody washout and with the experimental receptor expression levels (Table 1). The model simulation results (lines) are compared to the equivalent experimental data (points), and each optimized parameter set is represented with a separate line. Panels are separated by the normalization basis used, with “BS1” referring to simulations and experimental data normalized against the bound BS1 concentration in all cell lines and “Ab” referring simulations and experimental data where each antibody was normalized against itself. Each panel depicts all optimal parameter sets obtained with that particular normalization method. Model output and experimental data are each normalized to the output/data from the concentrations where binding reached saturation. The error bars depict the standard error from three experimental replicates. (PDF) [file pcbi.1012157.s011.pdf]

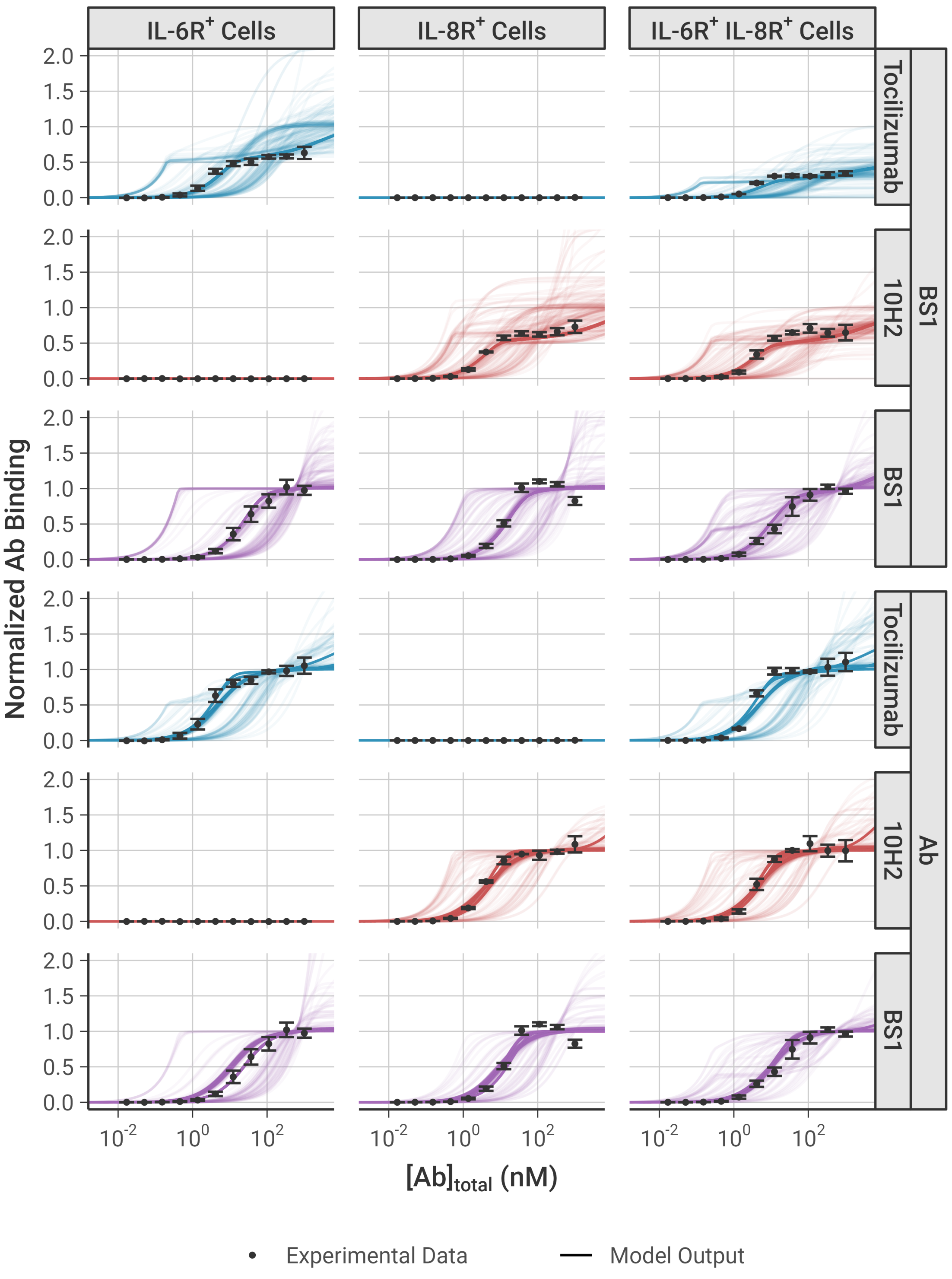


**S7 Fig. Model simulation results using each of the optimized parameter sets compared to the experimental data used to fit the model parameters.** Simulations were performed under the same conditions as the experiment, with a 2-hour initial binding period followed by a 15-minute antibody washout and with the experimental receptor expression levels [**Table 1**]. The model simulation results (lines) are compared to the equivalent experimental data (points), and each optimized parameter set is represented with a separate line. Panels are separated by the normalization basis used, with “BS1” referring to simulations and

experimental data normalized against the bound BS1 concentration in all cell lines and “Ab” referring simulations and experimental data where each antibody was normalized against itself. Each panel depicts all optimal parameter sets obtained with that particular normalization method. Model output and experimental data are each normalized to the output/data from the concentrations where binding reached saturation. The error bars depict the standard error from three experimental replicates.
