# Supplementary material for: Mechanistic computational modeling of monospecific and bispecific antibodies targeting interleukin-6/8 receptors
Source: PLoS Comput Biol. 2024 Jun 7;20(6):e1012157. doi: 10.1371/journal.pcbi.1012157 (PMC11189202; doi:10.1371/journal.pcbi.1012157)
Supplement: S8 Fig — Cell number = 1 × 10^5^ for all simulations. Free antibody concentration was set to 0 nM at 2 hours to simulate antibody washout from the system. The expressions of IL-6R and IL-8R from the transduced experimental cell lines were used in the simulations (Table 1). A, Simulations with an initial BS1 concentration of 10 nM, compared to the simulations with 100 nM of BS1 shown in the main text (Fig 4). B, Simulations of tocilizumab at an initial concentration of 100 nM in the IL-6R+ cell lines. C, Simulations of 10H2 at an initial concentration of 100 nM in the IL-8R+ cell lines. (PDF) [file pcbi.1012157.s012.pdf]

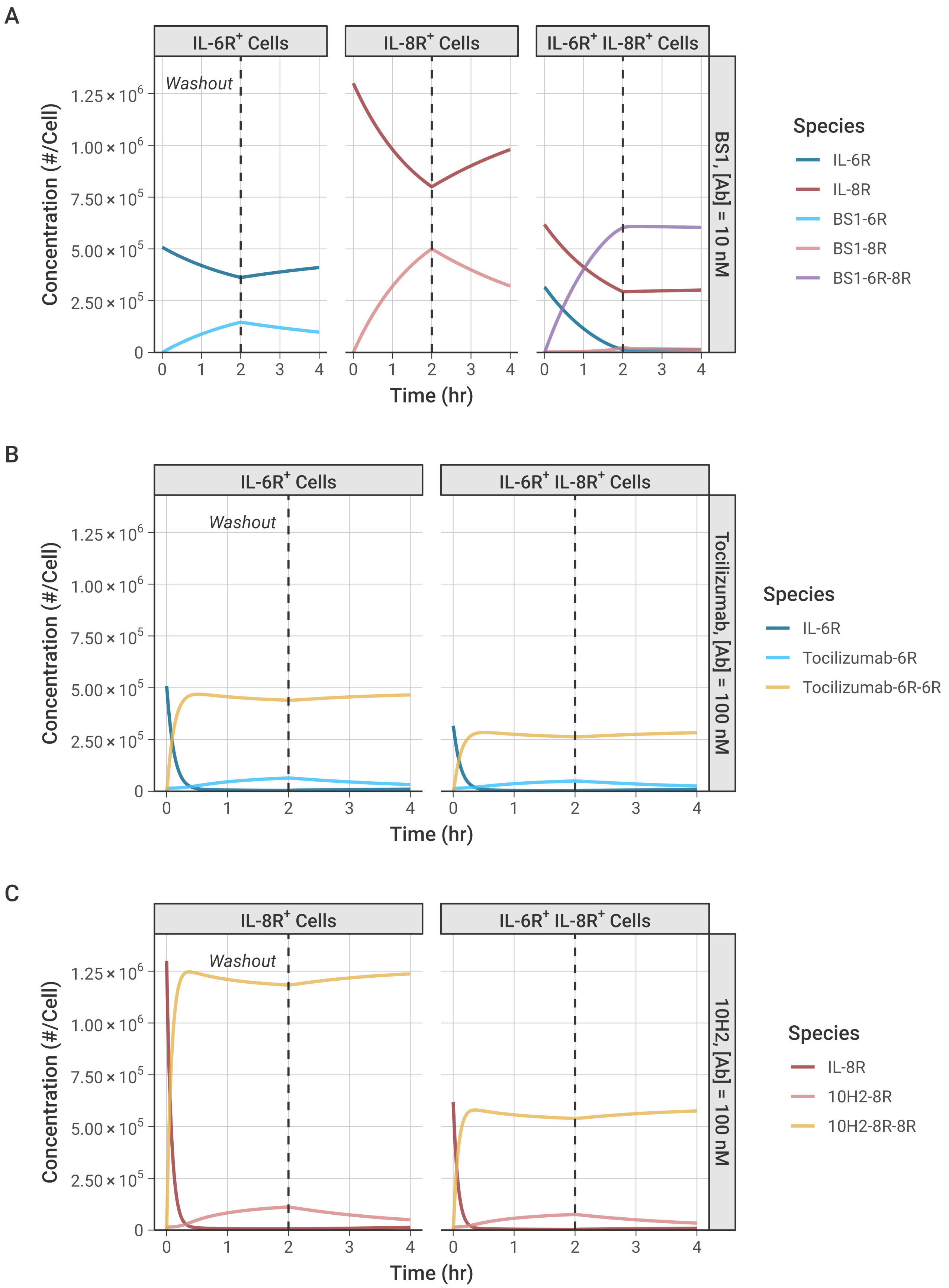
A
IL-6R+ Cells
IL-8R+ Cells
IL-6R+ IL-8R+ Cells
Washout
BS1, [Ab] = 10 nM
Concentration (#/Cell)
1.25 × 10^6
1.00 × 10^6
7.50 × 10^5
5.00 × 10^5
2.50 × 10^5
0
0 1 2 3 4
Time (hr)
Species
IL-6R
IL-8R
BS1-6R
BS1-8R
BS1-6R-8R
B
IL-6R+ Cells
IL-6R+ IL-8R+ Cells
Washout
Tocilizumab, [Ab] = 100 nM
Concentration (#/Cell)
Time (hr)
Species
IL-6R
Tocilizumab-6R
Tocilizumab-6R-6R
C
IL-8R+ Cells
IL-6R+ IL-8R+ Cells
Washout
10H2, [Ab] = 100 nM
Concentration (#/Cell)
Time (hr)
Species
IL-8R
10H2-8R
10H2-8R-8R

S8 Fig. Simulations quantify bivalent antibody binding to IL-6R and IL-8R over time. Cell number = 1 × 10^5 for all simulations. Free antibody concentration was set to 0 nM at 2 hours to simulate antibody washout from the system. The expressions of IL-6R and IL-8R from the transduced experimental cell lines were used in the simulations [**Table 1**]. **A,** Simulations with an initial BS1 concentration of 10 nM, compared to the simulations with 100 nM of BS1 shown in the main text [**Fig 4**]. **B,** Simulations of tocilizumab at an initial concentration of 100 nM in the IL-6R$^+$ cell lines. **C,** Simulations of 10H2 at an initial concentration of 100 nM in the IL-8R$^+$ cell lines.
