# Supplementary material for: Mechanistic computational modeling of monospecific and bispecific antibodies targeting interleukin-6/8 receptors
Source: PLoS Comput Biol. 2024 Jun 7;20(6):e1012157. doi: 10.1371/journal.pcbi.1012157 (PMC11189202; doi:10.1371/journal.pcbi.1012157)
Supplement: S9 Fig — IL-6R and IL-8R are present in a 1:1 ratio, as are tocilizumab and 10H2. Simulations were performed for 24 hours after antibody dosing. The color indicates the fraction of the total receptor (IL-6R + IL-8R) that is bound in each antibody-receptor complex type. (PDF) [file pcbi.1012157.s013.pdf]

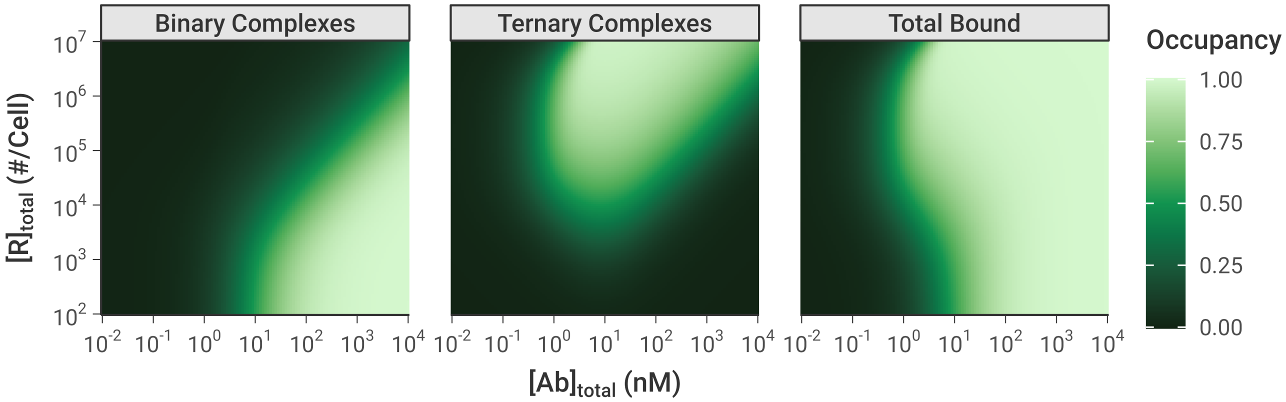

S9 Fig. Simulated Binary (Ab-R), Ternary (R-Ab-R), and Total Bound (Binary + Ternary) concentrations of Antibody-Receptor complexes using the combination of tocilizumab and 10H2. IL-6R and IL-8R are present in a 1:1 ratio, as are tocilizumab and 10H2. Simulations were performed for 24 hours after antibody dosing. The color indicates the fraction of the total receptor (IL-6R + IL-8R) that is bound in each antibody-receptor complex type.
