# Supplementary material for: Mechanistic computational modeling of monospecific and bispecific antibodies targeting interleukin-6/8 receptors
Source: PLoS Comput Biol. 2024 Jun 7;20(6):e1012157. doi: 10.1371/journal.pcbi.1012157 (PMC11189202; doi:10.1371/journal.pcbi.1012157)
Supplement: S10 Fig — Fraction of total BS1 and receptor concentrations free and bound over varying initial BS1 concentration. The first panel shows the fraction of total BS1 concentration that is unbound, and the other panels show the fraction of total receptor concentration (IL-6R + IL-8R) that is unbound and bound. The association rate constants for the formation of ternary complexes (kon,6R* and kon,8R*) were set to 0 to restrict BS1 to monovalent binding only. IL-6R and IL-8R are present in a 1:1 ratio, and simulations were performed for 24 hours after antibody dosing. (PDF) [file pcbi.1012157.s014.pdf]

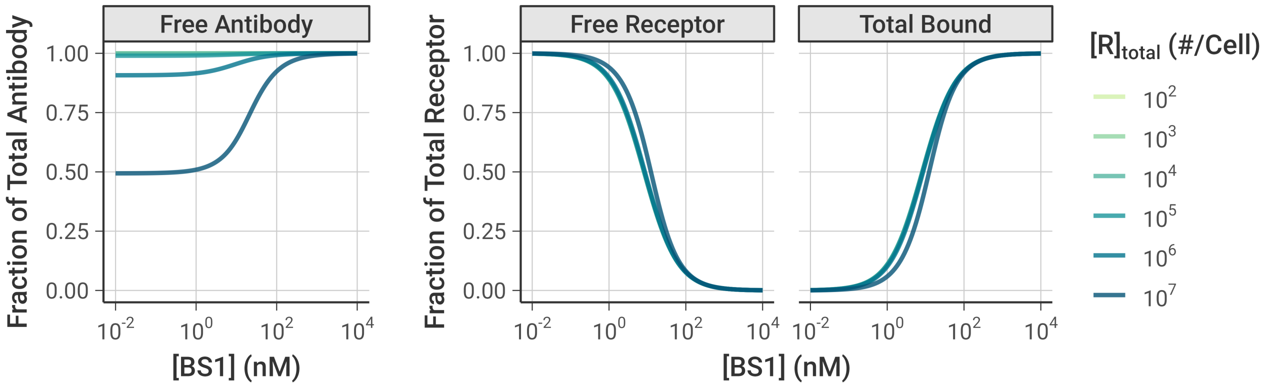

S10 Fig. Simulations of monovalent BS1 binding. Fraction of total BS1 and receptor concentrations free and bound over varying initial BS1 concentration. The first panel shows the fraction of total BS1 concentration that is unbound, and the other panels show the fraction of total receptor concentration (IL-6R + IL-8R) that is unbound and bound. The association rate constants for the formation of ternary complexes ($k_{on,6R^*}$ and $k_{on,8R^*}$) were set to 0 to restrict BS1 to monovalent binding only. IL-6R and IL-8R are present in a 1:1 ratio, and simulations were performed for 24 hours after antibody dosing.
