# Supplementary material for: Mechanistic computational modeling of monospecific and bispecific antibodies targeting interleukin-6/8 receptors
Source: PLoS Comput Biol. 2024 Jun 7;20(6):e1012157. doi: 10.1371/journal.pcbi.1012157 (PMC11189202; doi:10.1371/journal.pcbi.1012157)
Supplement: S11 Fig — Simulations were performed for over varying initial antibody and receptor concentrations. The association rate constants for the formation of ternary complexes (kon,6R* and kon,8R*) were set to 0 to restrict both antibodies to monovalent binding only. IL-6R and IL-8R are present in a 1:1 ratio, as were tocilizumab and 10H2, and simulations were performed for 24 hours after antibody dosing. The simulation conditions are the same as those shown for BS1 in the main text (Fig 6). A, Fraction of total antibody concentration (tocilizumab + 10H2) that is free (unbound) for different levels of receptor expression and initial antibody concentration. B, Fraction of total receptor concentration (IL-6R + IL-8R) that is unbound (free) or bound (in binary antibody-receptor complexes) for different levels of receptor expression and initial total antibody (tocilizumab + 10H2) concentration. C, Heat map of bound receptor fraction over varying antibody and receptor concentrations. The color indicates the fraction of the total receptor (IL-6R + IL-8R) that is bound to antibody. D, Comparison of monovalent and bivalent binding. The lines indicate the fraction of total receptor (IL-6R + IL-8R) that is bound in different complex types in the original simulations and the simulations restricted to monovalent binding only. The panels are divided by the total receptor concentration (in # receptors/cell). (PDF) [file pcbi.1012157.s015.pdf]

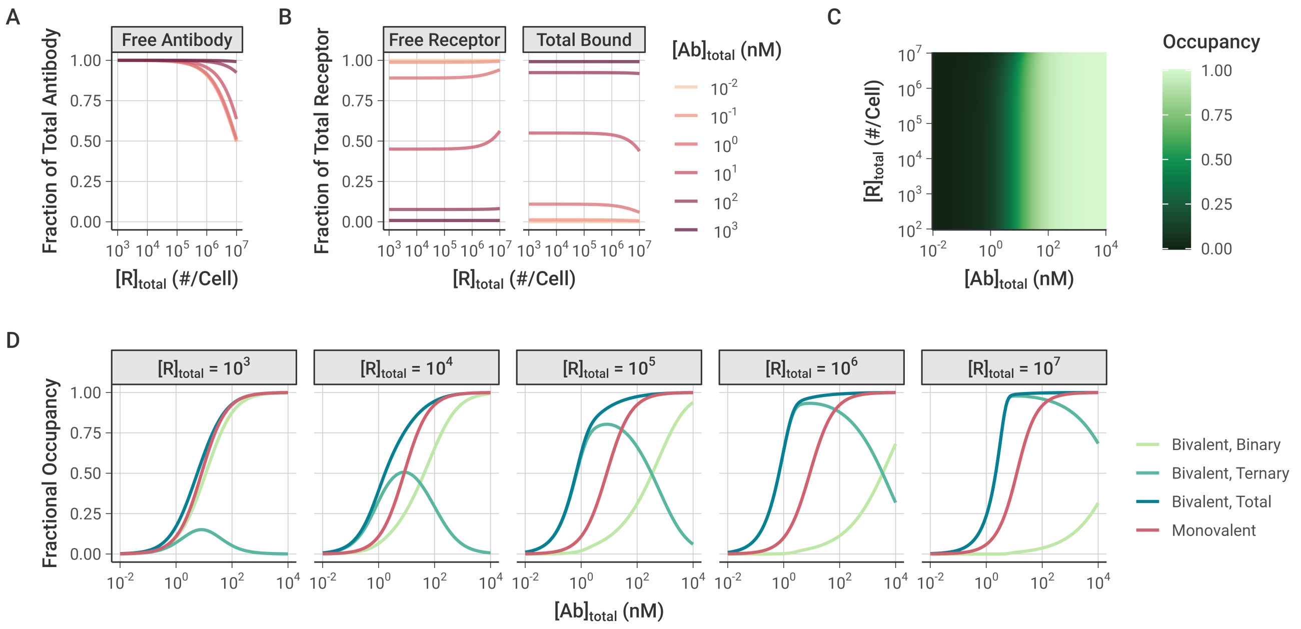

S11 Fig. Simulations of the combination of tocilizumab and 10H2 restricted to monovalent binding only. Simulations were performed for over varying initial antibody and receptor concentrations. The association rate constants for the formation of ternary complexes ($k_{on,6R^*}$ and $k_{on,8R^*}$) were set to 0 to restrict both antibodies to monovalent binding only. IL-6R and IL-8R are present in a 1:1 ratio, as were tocilizumab and 10H2, and simulations were performed for 24 hours after antibody dosing. The simulation conditions are the same as those shown for BS1 in the main text [**Fig 6**]. **A,** Fraction of total antibody concentration (tocilizumab + 10H2) that is free (unbound) for different levels of receptor expression and initial antibody concentration. **B,** Fraction of total receptor concentration (IL-6R + IL-8R) that is unbound (free) or bound (in binary antibody-receptor complexes) for different levels of receptor expression and initial total antibody (tocilizumab + 10H2) concentration. **C,** Heat map of bound receptor fraction over varying antibody and receptor concentrations. The color indicates the fraction of the total receptor (IL-6R + IL-8R) that is bound to antibody. **D,** Comparison of monovalent and bivalent binding. The lines indicate the fraction of total receptor (IL-6R + IL-8R) that is bound in different complex types in the original simulations and the simulations restricted to monovalent binding only. The panels are divided by the total receptor concentration (in # receptors/cell).
