# Supplementary material for: Mechanistic computational modeling of monospecific and bispecific antibodies targeting interleukin-6/8 receptors
Source: PLoS Comput Biol. 2024 Jun 7;20(6):e1012157. doi: 10.1371/journal.pcbi.1012157 (PMC11189202; doi:10.1371/journal.pcbi.1012157)
Supplement: S12 Fig — The color indicates the fraction of total receptor (IL-6R + IL-8R) that is bound in each antibody-receptor complex type. The antibody concentration is the total initial concentration of antibody in the system; “mAbs” refers to tocilizumab and 10H2 together in a 1:1 concentration ratio. Simulations were performed for 24 hours after antibody dosing. The panels for [Ab] = 10 nM were presented in the main text (Fig 7A) and are repeated here for comparison to the other concentrations. (PDF) [file pcbi.1012157.s016.pdf]

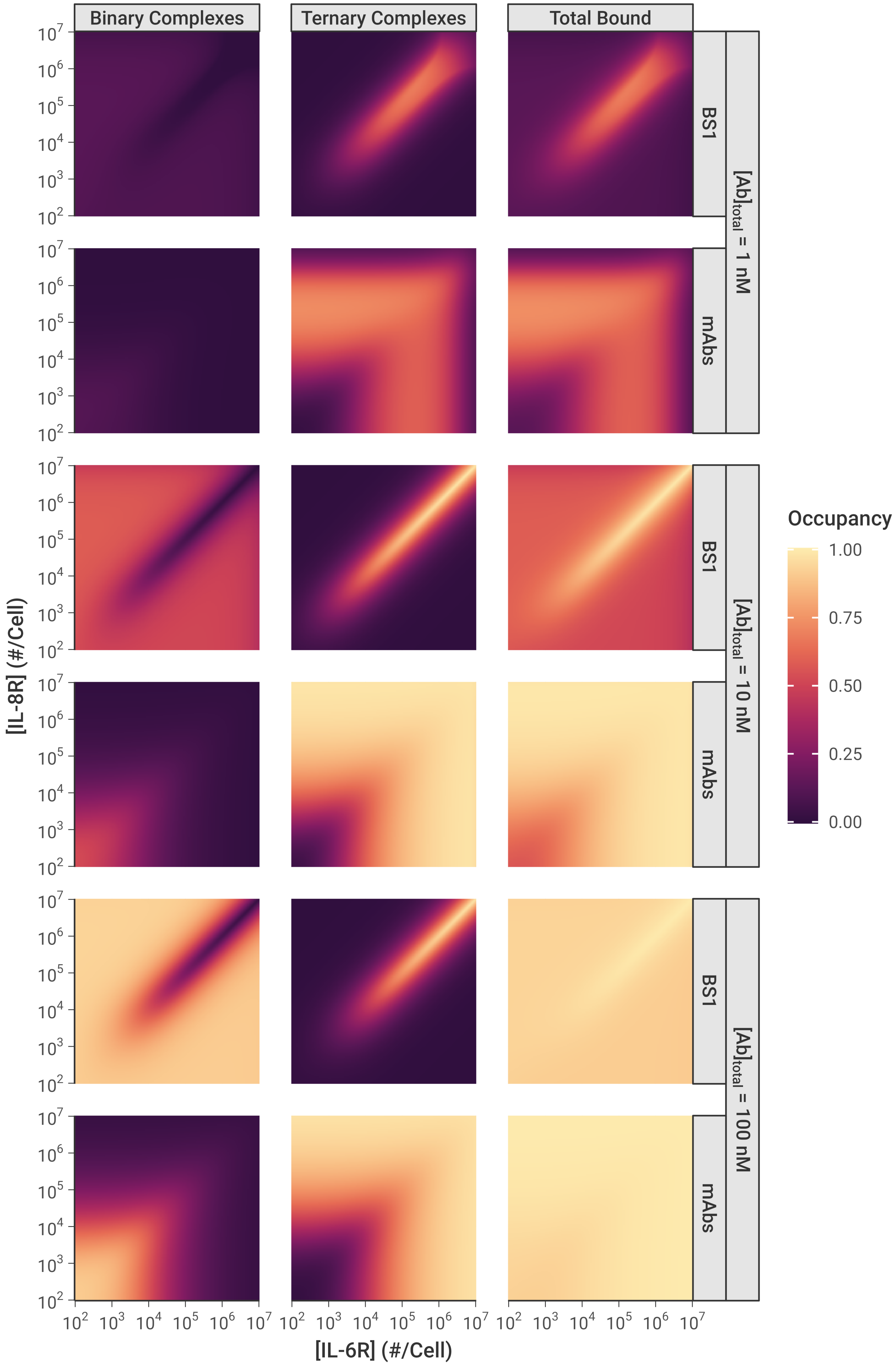

Binary Complexes
Ternary Complexes
Total Bound
BS1
mAbs
[Ab]total = 1 nM
[Ab]total = 10 nM
[Ab]total = 100 nM
Occupancy
1.00
0.75
0.50
0.25
0.00
[IL-8R] (#/Cell)
[IL-6R] (#/Cell)
10^2
10^3
10^4
10^5
10^6
10^7

S12 Fig. Fraction of receptors bound in Binary and Ternary complexes and Total Bound receptor (Binary + Ternary) across different IL-6R and IL-8R expression levels and initial antibody concentrations. The color indicates the fraction of total receptor (IL-6R + IL-8R) that is bound in each antibody-receptor complex type. The antibody concentration is the total initial concentration of antibody in the system; “mAbs” refers to tocilizumab and 10H2 together in a 1:1 concentration ratio. Simulations were performed for 24 hours after antibody dosing. The panels for [Ab] = 10 nM were presented in the main text [**Fig 7A**] and are repeated here for comparison to the other concentrations.
