# Supplementary material for: Mechanistic computational modeling of monospecific and bispecific antibodies targeting interleukin-6/8 receptors
Source: PLoS Comput Biol. 2024 Jun 7;20(6):e1012157. doi: 10.1371/journal.pcbi.1012157 (PMC11189202; doi:10.1371/journal.pcbi.1012157)
Supplement: S13 Fig — IL-8R was fixed at 103 receptors/cell for these simulations, while IL-6R ranged from 102 to 107 receptors/cell. The fractional occupancy indicates the fraction of the specific receptor concentration (either IL-6R or IL-8R) that is bound to antibody (either BS1 or the combination of tocilizumab and 10H2). The fractional occupancy when IL-6R was fixed and IL-8R was in excess was shown in the main text (Fig 7). (PDF) [file pcbi.1012157.s017.pdf]

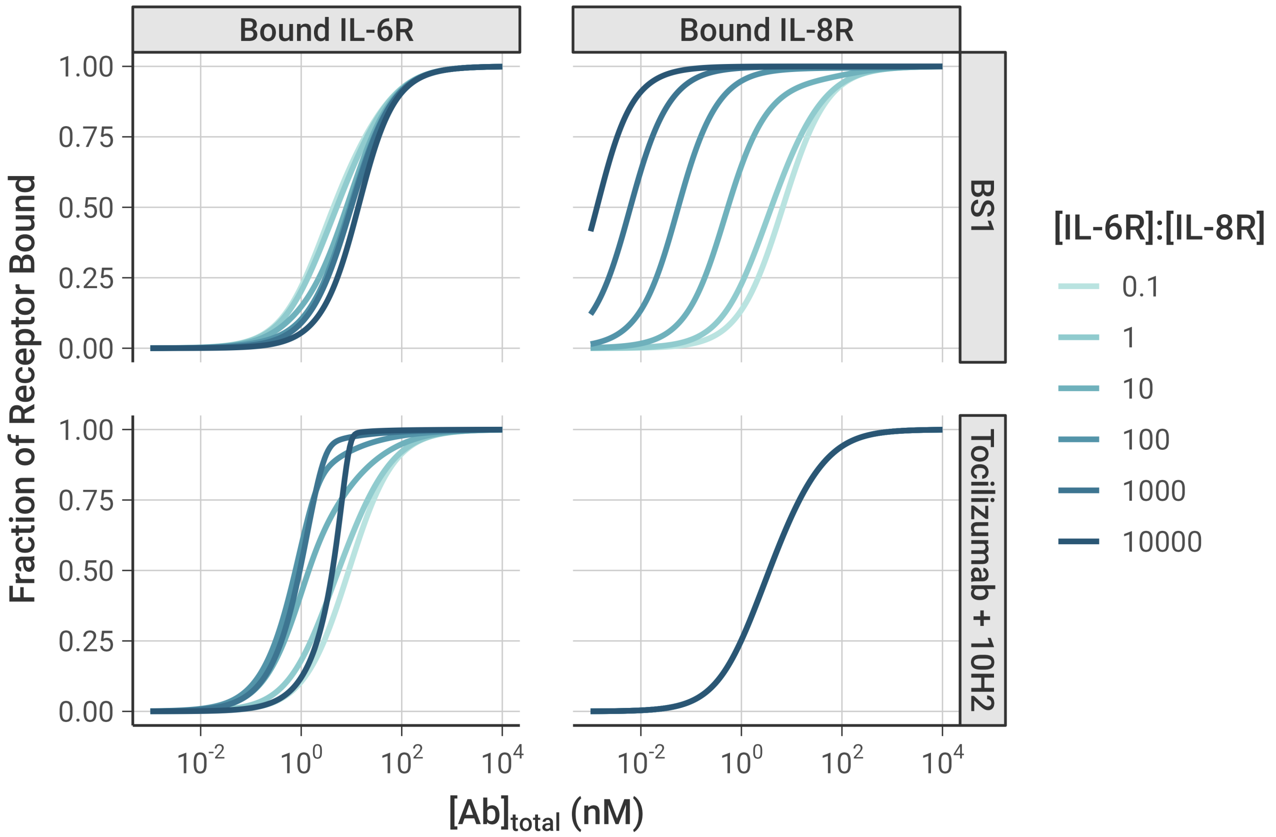

S13 Fig. The fractional occupancy of each receptor individually when one receptor (IL-6R) is in excess. IL-8R was fixed at 10^3 receptors/cell for these simulations, while IL-6R ranged from 10^2 to 10^7 receptors/cell. The fractional occupancy indicates the fraction of the specific receptor concentration (either IL-6R or IL-8R) that is bound to antibody (either BS1 or the combination of tocilizumab and 10H2). The fractional occupancy when IL-6R was fixed and IL-8R was in excess was shown in the main text [**Fig 7**]
