# Supplementary material for: Mechanistic computational modeling of monospecific and bispecific antibodies targeting interleukin-6/8 receptors
Source: PLoS Comput Biol. 2024 Jun 7;20(6):e1012157. doi: 10.1371/journal.pcbi.1012157 (PMC11189202; doi:10.1371/journal.pcbi.1012157)
Supplement: S14 Fig — The color indicates the relative bound receptor, where relative binding is the ratio of fractional bound receptor (the fraction of total IL-6R + IL-8R bound to antibody) when BS1 is used compared to when the combination of mAbs is used. The antibody concentration is the total initial concentration of antibody in the system; “mAbs” refers to tocilizumab and 10H2 together in a 1:1 concentration ratio. Simulations were performed for 24 hours after antibody dosing. The panels for [Ab] = 10 nM were presented in the main text (Fig 7C) and are repeated here for comparison to the other concentrations. (PDF) [file pcbi.1012157.s018.pdf]

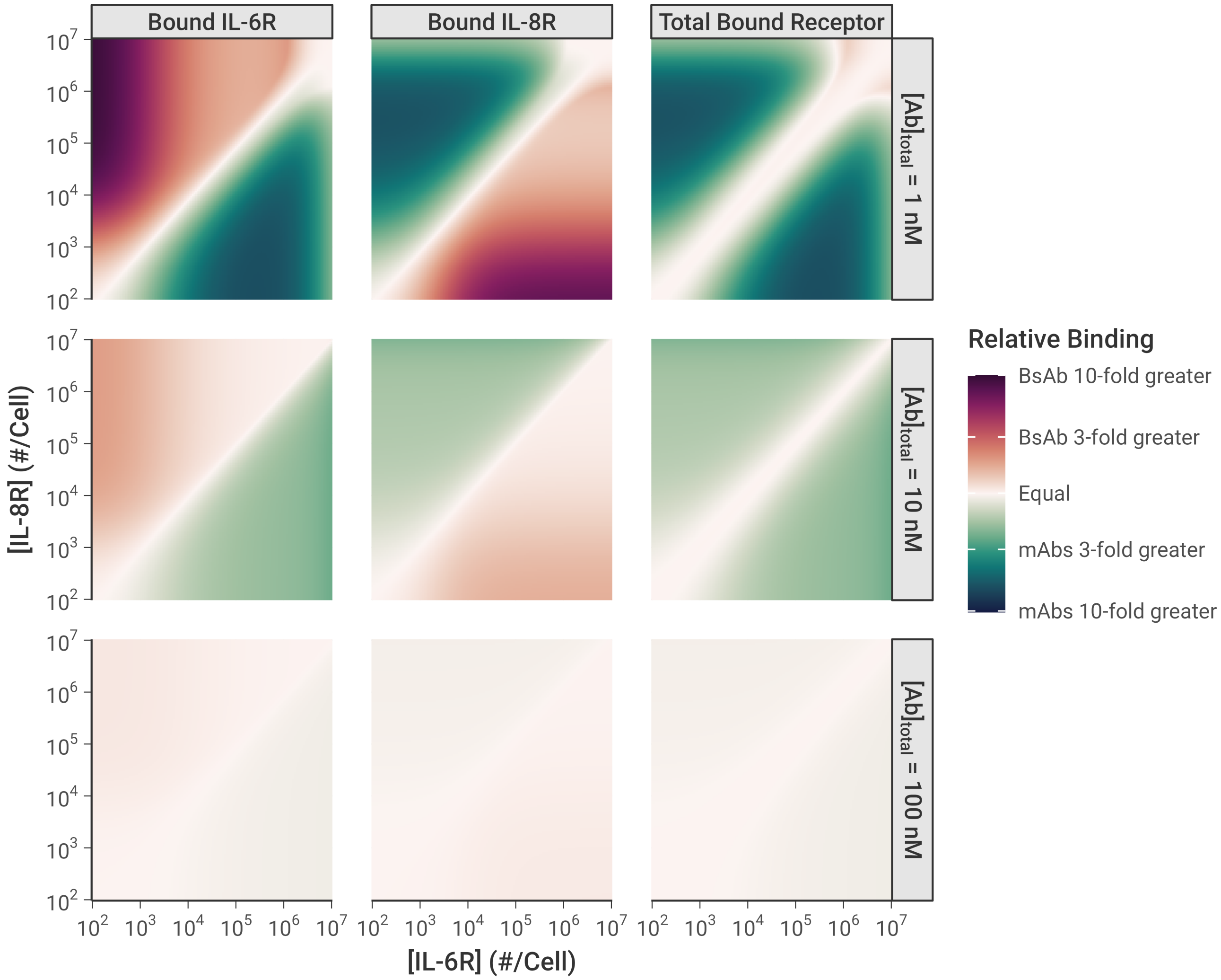

S14 Fig. Relative binding of BS1 and the combination of monoclonal antibodies across different IL-6R and IL-8R expression levels and initial total antibody concentrations. The color indicates the relative bound receptor, where relative binding is the ratio of fractional bound receptor (the fraction of total IL-6R + IL-8R bound to antibody) when BS1 is used compared to when the combination of mAbs is used. The antibody concentration is the total initial concentration of antibody in the system; “mAbs” refers to tocilizumab and 10H2 together in a 1:1 concentration ratio. Simulations were performed for 24 hours after antibody dosing. The panels for [Ab] = 10 nM were presented in the main text [**Fig 7C**] and are repeated here for comparison to the other concentrations.
